# Manual measurement of angles in backscattered and transmission Kikuchi diffraction patterns

**DOI:** 10.1107/S1600576720000692

**Published:** 2020-03-25

**Authors:** Gert Nolze, Tomasz Tokarski, Grzegorz Cios, Aimo Winkelmann

**Affiliations:** a Federal Institute for Materials Research and Testing (BAM), Unter den Eichen 87, 12205 Berlin, Germany; b Academic Centre for Materials and Nanotechnology, AGH University of Science and Technology, Mickiewicza 30, 30-059 Krakow, Poland

**Keywords:** electron backscatter diffraction, EBSD, angle measurement, gnomonic projections, Kikuchi patterns, Hilton net

## Abstract

A historical tool for crystallographic analysis is provided by the Hilton net, which can be used for manually surveying the crystal lattice as it is manifested by the Kikuchi bands in a gnomonic projection. For a quantitative analysis using the Hilton net, the projection centre as the relative position of the signal source with respect to the detector plane needs to be known. Interplanar angles are accessible with a precision and accuracy which is estimated to be ≤0.3°. Angles between any directions, *e.g.* zone axes, are directly readable. Finally, for the rare case of an unknown projection-centre position, its determination is demonstrated by adapting an old approach developed for photogrammetric applications. It requires the indexing of four zone axes [*uvw*]_*i*_ in a backscattered Kikuchi diffraction pattern of a known phase collected under comparable geometric conditions.

## Abstract 2

Procedures are demonstrated which enable a manual determination of the angles between lattice planes and directions from electron backscatter diffraction patterns of unknown phases. The determination of the projection centre is described for cases where it is unknown.

## 1. Introduction   

### 1.1. Full automation: a blessing and a curse   

In the technique of electron backscatter diffraction (EBSD), the processing and interpretation of backscattered Kikuchi diffraction (BKD) and transmission Kikuchi diffraction (TKD) patterns have been fully automated for many years. Commercial EBSD software is highly efficient at discriminating phases from a given fixed list of crystalline structures via detection and interpretation of Kikuchi bands, if their BKD signals are sufficiently different. The band detection is commonly performed by an automated search of individual peaks in Hough space (Krieger Lassen, 1994 ▸). However, during peak search the crystallographic inter­relations are completely ignored (Nolze & Winkelmann, 2017 ▸). In consequence, a fully automated phase discrimination reaches its limits if the phases generate comparable Hough peak distributions, or if they are not included in available databases or are unknown altogether. An optimization of the search procedure by variation of peak detection parameters may improve the discrimination between phases, but any interpretation of unknown phases remains an unsolved problem.

### 1.2. Need for manual pattern interpretation   

A professional assessment of unknown phases could partly be done by a manual analysis of single BKD patterns. A significant obstacle, however, is the lack of a tool for measuring the angles between diffracting lattice planes and/or directions. Although such angles can deliver first indications for the crystal symmetry or the description and identification of the translation lattice, appropriate measurement tools are not generally available.

An essentially similar task is the labelling of lattice directions or planes in single BKD patterns without the help of vendor-specific indexing software. For an unequivocal identification or verification, knowledge of the angles between such features is very beneficial.

Therefore, the present work demonstrates alternative ways of manually determining angles in BKD patterns. For such measurements, some (nonzero) value for the position of the signal source with respect to the detecting screen (termed the projection centre, denoted PC) must be provided. This information is typically known, but where this is not the case, a procedure is given which enables the calculation of this projection-centre position.

### 1.3. Crystallographic fundamentals   

When dealing with EBSD, it is crucial to realize that both lattice and structure information for a crystalline phase are encoded in a single BKD pattern [see *e.g.* Dingley & Wright (2009 ▸), Wright *et al.* (2009 ▸), Lühr *et al.* (2016 ▸) and Nolze *et al.* (2018 ▸)]. As for any other physical or chemical crystal property, BKD patterns have to follow crystallographic laws. Applied appropriately, they can help to draw lattice-plane traces and zone axes that are much more accurate than those extracted automatically by the currently implemented Hough peak-search method.

These EBSD-relevant fundamentals in geometric crystallography have been known for a long time. They include Steno’s law of constant angles (Stenonis, 1669 ▸), Haüy’s law of rational indices (Haüy, 1784 ▸), the Weiss law of assembly of zones (Weiss, 1814–1815 ▸) and Goldschmidt’s law of complication (Goldschmidt, 1897*a* ▸,*b* ▸). It should be obvious that an EBSD analysis can only benefit if these fundamentals are fully considered in the underlying algorithms, *e.g.* during Kikuchi band detection.

For the present paper, the relevant basics of a crystal lattice are so universal that they are even independent of symmetry and lattice parameters and therefore particularly valuable:

(i) A certain lattice plane (*hkl*) contains an infinite number of lattice directions [*uvw*]_*i*_ which are defined by
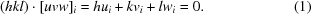



(ii) Two lattice directions always describe a lattice plane,
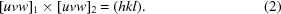



(iii) The intersection of two lattice planes is always a lattice direction,
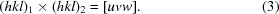



For BKD patterns representing the azimuthal projection of diffraction phenomena from waves of the same wavelength with a crystal lattice, certain consequences result:

(i) The crossing point of two (non-identified) lattice-plane traces defines the intersection point of an (unknown) lattice direction [*uvw*] with the projection screen.

(ii) Conversely, the connection between two lattice directions always defines a trace of a lattice plane (*hkl*), regardless of whether this plane actually forms a visibly strong Kikuchi band or not.

Moreover, the most relevant specific properties for a spherical projection of a crystal lattice are the following:

(i) The intersection of a lattice plane with the projection sphere defines a great circle.

(ii) The intersection of a Kossel cone with the projection sphere (Kikuchi band edge) is, however, a small circle.

(iii) The shortest distance between two points on a sphere is given along the shared great circle. It represents the angle between the two vectors defined by the centre of the sphere and the two points.

(iv) The position of the source volume is identical to the centre of the projection sphere. It is therefore called the projection-centre (PC) position. PC = [PC_*x*_, PC_*y*_, PC_*z*_] is here defined in terms of the image coordinate system.

The historical significance of the gnomonic projection for crystallography is discussed, for example, by Miller (1859 ▸), Hilton (1905 ▸), Smith (1919 ▸) and Dijkstra (1949 ▸). For the interpretation of BKD patterns some crucial properties are important:

(i) In the gnomonic projection a (lattice) plane always becomes a straight line, which is the reason why the Hough transformation is used for automated band detection.

(ii) The position of the pattern centre PC′ is a subset of PC. It indicates the ‘point of no distortion’ and can be described differently, *e.g.* regarding the PC as

(*a*) a perpendicular projection of PC on the projection plane, *i.e.* PC′ = [PC_*x*_, PC_*y*_, 0] 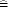 [PC_*x*_, PC_*y*_], or as

(*b*) a projected point with the shortest distance to PC.

Alternatively, characteristic properties of the projection can be used. Thus, PC′ is also given by

(*c*) the intersection point of the main axes of (elliptic) higher-order Laue zone (HOLZ) rings,

(*d*) the intersection point of all band normals fixed at the position of the respective smallest band width, or

(*e*) the projection point of a (crystal) direction which is invariant during movement of the detection screen.

Finally, well known properties of the visualized object need to be correctly projected. For crystals this concerns

(*f*) the very characteristic angles between lattice planes and directions. Correct projection only results if PC and therefore also PC′ are perfectly described. This approach is applied in *EBSDL* (Li & Han, 2015 ▸) during simultaneous determination of the Bravais lattice type, lattice parameters and PC.

We will utilize the universal properties of crystals and projective geometry to demonstrate that the angles between lattice planes and directions in BKD or TKD patterns are accessible without specialized software. The precision of the manual approach presented here is in many cases sufficient for further phase analysis and discrimination.

## 2. Angle measurement in BKD patterns   

### 2.1. Band position and diffracting lattice plane   

The object of most EBSD measurements is the correct recognition of the diffracting phase and the derivation of the crystal orientation from BKD patterns. To this end, a suitable number of band-shaped intensity features are automatically detected. To a first approximation they are correlated with projections of diffracting lattice planes (*hkl*)_*i*_. Since for many phases the angles between (*hkl*)_*i*_ are characteristic (*cf*. Steno’s law), they are used during discrimination between a fixed set of phases.

**Figure 1:**
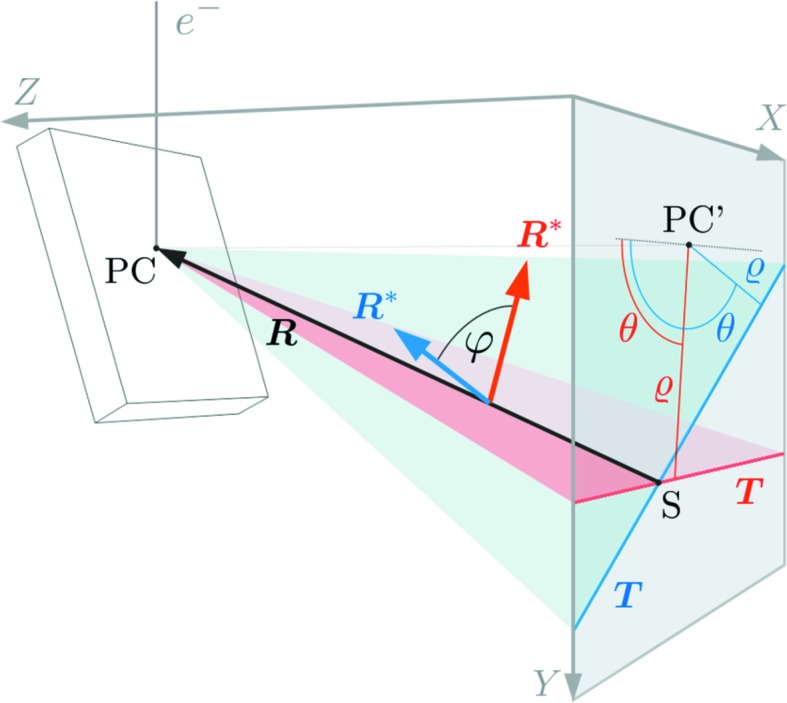
Alignment (slope θ and distance 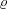 from the pattern centre PC′) of lattice-plane traces **T**_*i*_. φ describes the angle (essential for EBSD) between the lattice-plane normals 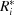 which result from the vector product **T**_*i*_ × **R**.

The principle of the applied angle determination is based on the assumption of a purely geometric projection model of lattice planes, as sketched in Fig. 1 ▸. In brief, lattice-plane traces are used in combination with the shared projection centre PC to define the relative inclination between (*hkl*)_*i*_.

Alternatively, the trace intersections can be used directly in order to identify lattice directions [*uvw*] which have to be parallel to **R** (Fig. 1 ▸). Angles between [*uvw*]_*i*_ are just as characteristic as angles between (*hkl*)_*i*_ but not quite as convenient to derive automatically.

### 2.2. Universal protractor for BKD patterns   

The necessary angular description of Kikuchi bands is nowadays generally performed via Hough or Radon transformation of a BKD pattern (Krieger Lassen, 1992 ▸). This operation transforms linear-shaped intensity accumulations into point-shaped maxima so that from an intensity maximum in Hough space the former band alignment can be deduced. A Hough peak encodes the slope θ and distance 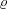 of a lattice-plane trace **T** to the circumference of a circular region of interest in the BKD pattern. If the projection centre PC is known, the alignment of the diffracting lattice plane can be unequivocally described by 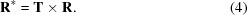

**R** is the crystal direction passing through PC and an intersection point *S* and is formed by the two^1^ concerned lattice planes (Fig. 1 ▸).

**Figure 2:**
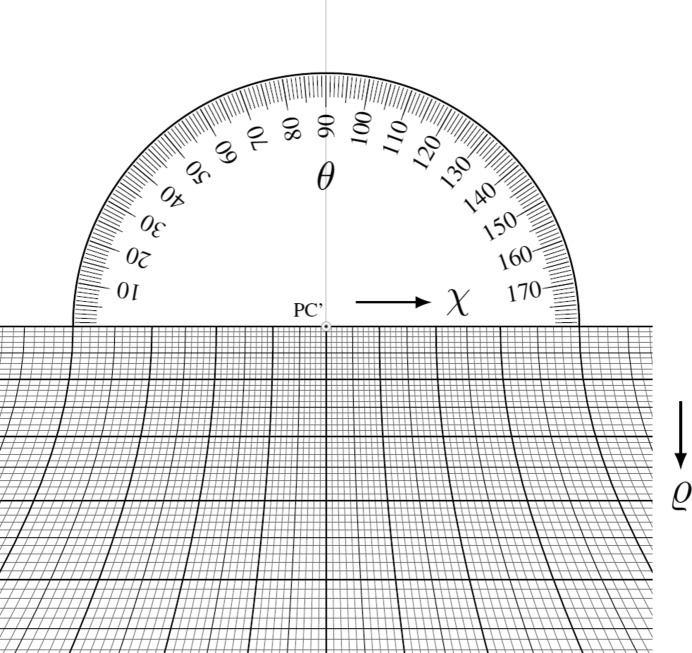
The Hilton net. It enables the measurement of the slope θ of a lattice-plane trace, and also its angular distance 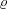 to the detection screen normal (marked PC′). Additionally, the angular distance χ between lattice directions along a lattice-plane trace can be measured. All angles are displayed with a step size of 1°.

A similar procedure to the Hough transformation can be performed manually. To this end, a protractor is helpful, and this approach was invented as the gnomonic net in crystallography more than a hundred years ago by Hilton (1904 ▸, 1905 ▸) (Fig. 2 ▸). This net, later named after its discoverer, represents an equatorial or meridional gnomonic net (Amorós *et al.*, 1975 ▸; Terpstra & Codd, 1961 ▸). However, it probably fell out of use after the discovery of X-ray diffraction in 1912 and the rapid spread of techniques that followed.

Nevertheless, the Hilton net represents the perfect tool for the convenient manual measurement of the slope θ of a lattice-plane trace and also of the tilt angle 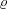 of the corresponding lattice plane against the detection screen normal. Additionally, along the great circles (horizontal straight lines) the equi­angular distance is given as the angle χ. Constant χ values form hyperbola arms, representing parts of equidistant small circles in the gnomonic projection. The resulting grid in the lower half of Fig. 2 ▸ can be easily calculated using
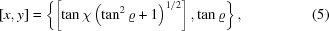

*i.e.* 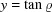 defines the distance of the great circles from the pattern centre PC′. However, since the traces of lattice planes are analysed, the angle between the normal vector of (*hkl*) and the pattern centre direction is given by 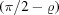.

### 2.3. Measurement and calculation of the angle between (*hkl*)   

#### 2.3.1. Size adjustment of the Hilton net   

Whereas the slope θ is independent of PC and PC′, 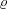 is greatly affected, which requires a calibration of the size of the Hilton net. This is comparatively straightforward since PC is known. The projection centre PC is given in relative terms, *i.e.* the absolute size of the detector screen and the real distance between the sample and detector are irrelevant. The reference dimension is the vertical size of the image (the number of pixels, or a corresponding size in millimetres) which is defined to be *D* = 1. In consequence, a projection centre described as PC = (0.5, 0.3, 0.8) defines a position of the source volume which has a distance to the present image of 0.8*D*. Along the horizontal image dimension the pattern centre is in the middle of the image.^2^ PC_*y*_ = 0.3 means that, depending on the manufacturer-dependent definition,^3^ the pattern centre is 30% away from the upper pattern edge.

For a proper scaling of the Hilton net, PC can be assumed to be at the upper edge of the pattern, *i.e.* PC_*y*_ = 0, so that the vertical reference solid angle 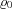 results:
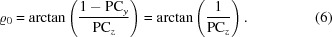
In order to generate a correct measurement of 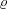 and χ, the sizes of the Hilton net and/or detector image must first be adapted by fixing the upper edge of the pattern at 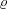 = 0, and enlarging the pattern or the net until the lower edge of the pattern exactly matches 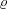 = 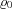. Second, for a real survey of 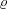 and χ, the Hilton net needs to be shifted until the net origin and the real PC′ in the pattern are located on top of each other.

Summarizing, in addition to the projection-centre position, the calibration of the Hilton net does not need a diffraction pattern at all but only any image collected from the camera displaying *e.g.* a background only. Also, the absolute size of the screen is irrelevant, which is quite beneficial since the physical size of the detecting screen and the image captured by the camera are commonly not identical.

#### 2.3.2. Alignment of lattice planes   

**Figure 3:**
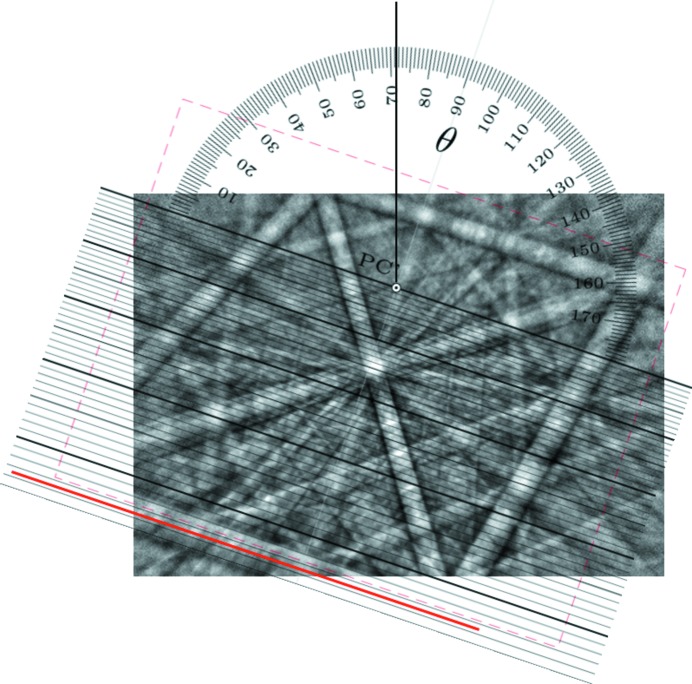
Measurement of the azimuth (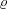 ≃ 71.4°) and polar angle (θ ≃ 43.7°) of a lattice-plane trace, highlighted by a bold red line. The dashed red frame indicates the size and position of the backscattered Kikuchi diffraction pattern for 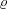 = θ = 0 . This initial alignment automatically defines the real position of PC′ in the pattern since it is indicated by the origin (rotation point) of the Hilton net. The experimental BKD pattern comes from magnetite (Fe_3_O_4_).

Fig. 3 ▸ shows an alternative way of adjusting the pattern centre size and the origins of, respectively, the Hilton net and the BKD pattern in a single step. This results in the dashed red frame (for θ = 0), which defines both the size (PC_*z*_) and position (PC_*x*_, PC_*y*_) of the pattern. The Hilton net or BKD pattern needs to be enlarged and shifted until the pattern fits exactly inside the drawn frame. Then the net origin and pattern centre are congruent so that, by rotation around the pinned PC′, 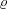 and θ can be directly measured for any lattice-plane trace (*cf.* the solid red line in Fig. 3 ▸).

The azimuth angle can be read from the common protractor which shows θ = 71.4 ± 0.1°.^4^ The polar angle between the screen normal and the lattice-plane trace is given by the distance of the straight line from PC′ and results in 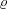 ≃ 43.7 ± 0.2°. Repeating this procedure, the alignment of each lattice-plane trace *i* can be unambiguously characterized by a set of 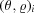. These are the preconditions for the determination of any angle φ_*i*, *j*_ between lattice planes *i* and *j*.

#### 2.3.3. Angle measurement   

Unfortunately, the angle between lattice planes cannot be measured directly by the Hilton net. A classic procedure is to enter all poles in a stereographic projection and measure the pole distance manually using the Wulff net. The angular distance between lattice-plane poles aligned on the shared great circle defines the interplanar angle. A detailed description of the usage of stereographic projection in crystallography is given, for example, by Terpstra & Codd (1961 ▸), Hammond (2009 ▸), Barrett (1937 ▸) and Johari & Thomas (1969 ▸).

Because of the limited precision during manual handling of the Wulff net and pole drawing, the uncertainty is typically in the region of 2°. With each additionally performed (manual) rotation of data the errors increase further. However, such manual procedures are nowadays easily replaceable by simple spreadsheets and do not require specialized software.

#### 2.3.4. Angle calculation: standard approach   

Default procedures for the determination of interplanar angles from EBSD patterns use the geometric relationships sketched in Fig. 1 ▸, *cf. e.g.* Wright & Adams (1992 ▸). The necessary definition of the lattice-plane traces may utilize a Hough or Radon transformation (Krieger Lassen, 1992 ▸; Schwarzer & Sukkau, 2003 ▸), but we can also use 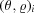 measured with the Hilton net.

Each trace **T**_*i*_ of a lattice plane (*hkl*)_*i*_ can be described by a linear equation using the measured angles,
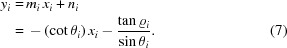
Inserting *x*_1_ = 0 and *x*_2_ = 1 in equation (7), **T**_*i*_ becomes an unexpectedly simple vector which only contains the slope of the trace,
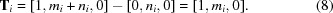
The lattice-plane normals 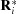 and 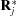 are given by the cross products between the trace **T** and the vector **R** connecting the intersection *S* of two traces with PC. This results in a direction which has the form
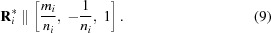
If 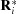 is calculated exactly as in expression (9), the required angle can be computed using
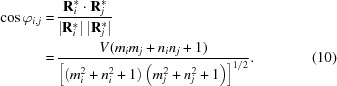
Note that the factor *V* = sign(*n_i_*) sign(*n_j_*) is essential to discriminate between φ and the complementary angle (π − φ).

While the explicit PC is absent from the formula, it is implicitly considered during scaling of the Hilton net and is therefore inherent in all 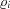, *i.e.* *n*_*i*_.

#### 2.3.5. Angle calculation simplified   

Actually, 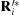 does not need to be derived via *m* and *n* since it can be computed directly from 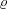 and θ:
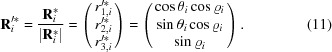
Since 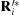 in equation (11) is the normalized description of vector 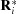 in (9), the calculation of φ_*i*, *j*_ in equation (10) simplifies to a scalar multiplication of vector coefficients:
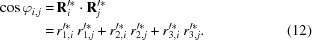
Unfortunately, such a procedure is not possible using the standard Hough transformation since 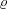 has to be derived first from PC and the roughly determined trace position.

#### 2.3.6. Example measurement   

Independent of the selected computation path, *i.e.* using either expression (10) or (12), for the strongest bands in the pattern in Fig. 3 ▸ the interplanar angles φ_*i*, *j*_ can be calculated (see Table 1 ▸). Here, only 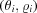 for the six strongest bands in the pattern of magnetite in Fig. 3 ▸ (Fe_3_O_4_, cubic, 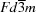) are considered. They are formed by {110} and {001}. Possible angles between symmetry-equivalent {110} are 60, 90 and 120°, whereas between {100} and {110} angles of 45, 90 and 135° may occur. It is pure coincidence that all these angles are listed in Table 1 ▸, inclusive of small errors resulting from the inaccurate measurement of θ and 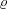. The maximum deviation of only ≤0.3° from the ideal angles has mainly three reasons:

(i) a nearly perfect PC position and thus an optimal scaling and alignment of the Hilton net,

(ii) the subtle definition of the lattice-plane traces, and

(iii) the semi-manual measurement of the angles 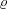 and θ.

The uncertainty in the PC position is minimized because the BKD pattern is from a cubic phase, which excludes any unforeseeable interferences from lattice-parameter ratios. Moreover, an extra pattern matching has been applied in order to obtain the most accurate PC description possible (Nolze & Winkelmann, 2017 ▸).

Regarding the trace description, the typically performed uncorrelated definition of lattice-plane traces commonly generates errors which are clearly higher than the small measurement error of θ and 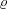 for such a line. The present state-of-the-art approach is a derivation of individual bands during a fully automated peak search in Hough space, or alternatively a manual drawing of lines as approximate centres of bands. As discussed in the *Introduction*, however, the trace positions are constrained by crystallographic fundamentals. From projective geometry it is well known that only four lattice-plane traces are required to derive further traces due to the connection of intersection points (Nolze & Winkelmann, 2017 ▸). New traces generate novel intersection points which can be used again to define further lattice-plane traces, and so on. The advantage of this approach is that misalignments of individually drawn traces are prevented. Any correction of a single line position is of a collective nature since all traces are connected to each other.

The precision of the manual angle measurement is estimated to be on the scale of Δθ ≤ 0.1° and 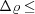 0.3°. Such small measurement errors were achievable because the spin animation in *PowerPoint* (Microsoft *Office* software) was used. This type of animation enables a very accurate evaluation of θ by the Hilton net.

Despite the half-automated spin animation, the angle measurement is still counted as a manual procedure. A more old-fashioned use of a Hilton net pinned and rotated on a printed BKD pattern would naturally be less correct. We assume values of 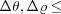 1°.

### 2.4. Angles between lattice directions   

#### 2.4.1. Angle measurement   

The Hilton net also enables a direct measurement of angles between directions. Since angular distances are in general measured along great circles, the straight lines in the Hilton net are used. Similar to the Wulff net for a stereographic projection of data, the Hilton net only needs to be rotated around the projection origin until both projection points share the same straight line.

**Figure 4:**
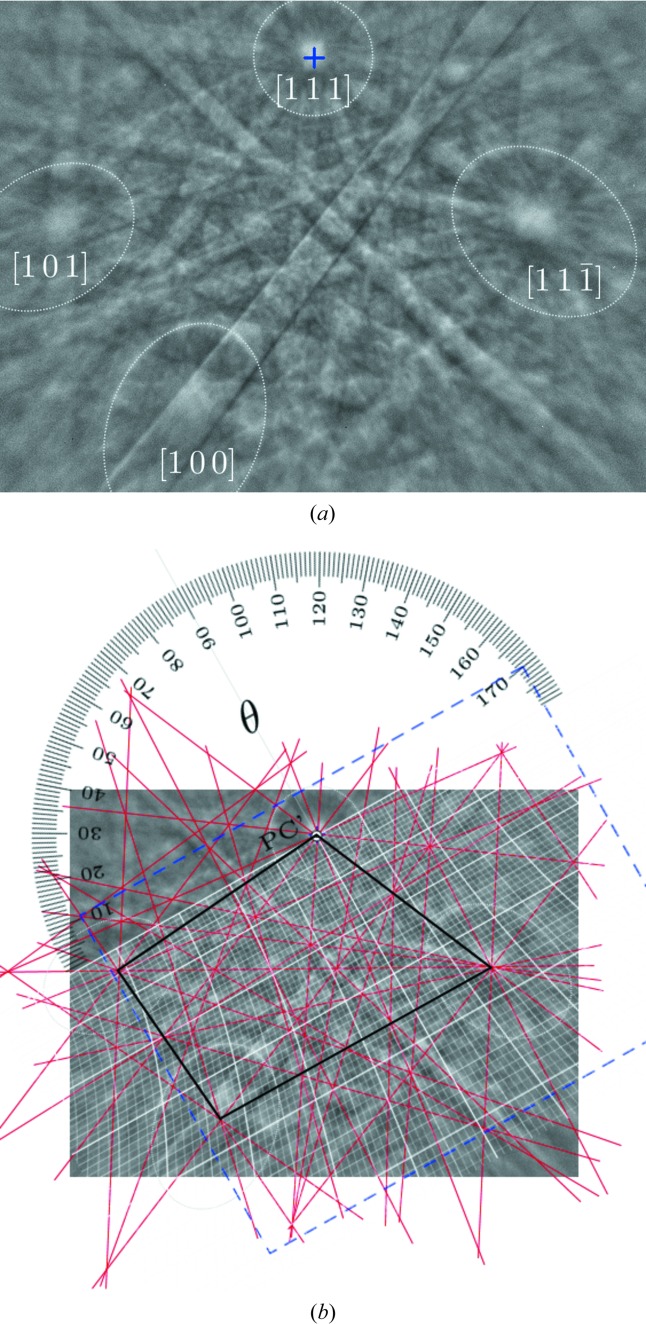
The angular distances between lattice directions defined by four axes of HOLZ rings [schreibersite, (Fe,Ni)_3_P]. (*a*) The blue cross marks the pattern centre PC′ as initially derived using the *Channel5* software (Oxford Instruments). (*b*) The dashed blue frame is used to calibrate the size of the Hilton net and consider the real position of PC′. The intersections of the lattice-plane traces (red lines) are used to define the correct positions of the wanted zone axes (corners of the black quadrangle). The Hilton net is aligned to enable measurement of the angle between two [*uvw*]_*i*_, yielding a value of ∼48°.

As an example, the BKD pattern in Fig. 4 ▸ is used. Fig. 4 ▸(*a*) shows HOLZ rings, which are highlighted by dotted lines in order to increase their visibility. The cone axis **R**_*i*_ of a HOLZ ring is defined by a lattice vector **R**_*i*_ ≡ [*uvw*]_*i*_ [*cf. e.g.* Michael & Eades (2000 ▸) and Nolze & Winkelmann (2017 ▸)], but its projected position is often quite vague. Therefore, in Fig. 4 ▸(*b*) lattice-plane traces are indicated as red lines because their crossing points define the required zone-axis positions. Note that the applied drawing procedure also defines zone-axis positions outside the captured BKD patterns which can be used as well. In particular, a manual measurement of a longer distance between poles results in a higher precision of the measured angle.

The angles measured between the four HOLZ-ring axes are compared with theoretical values calculated from the lattice parameters (Table 2 ▸). The values show a satisfying coincidence between measured and expected angles. They also suggest that the measurement errors are comparable to those reported for the lattice-plane angles. However, since for the definition of two zone axes four lattice planes are involved, the error is assumed to be slightly higher, Δχ < 0.5°.

Since the Hilton net enables angular measurements between any two directions, a number of possible applications can be discussed. For instance, the solid angle covered by the screen used is measurable, band widths can at least be classified, or the opening angles of the different HOLZ rings in Fig. 4 ▸(*a*) can be measured and compared. For the last example, the (actually invisible) zone axis needs to be on the great circle since only then is the real diameter of the HOLZ ring defined and the angle measured correctly. Such investigations confirm that the ellipses formed by HOLZ phenomena are, in fact, circles on a sphere. Independent of the selected 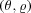 but with the zone axis on the used great circle, the same χ (diameter) results. The two non-equivalent HOLZ rings along [111] and [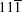] for schreibersite (space group 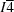) have a diameter of 19.5°, whereas for [101] and [100] their diameters are 16.5 and 16°, *i.e.* considerably smaller.

## 3. Manual determination of the projection centre PC   

For all previously discussed applications of the Hilton net the position of PC was preconditional. We have shown that PC determines the size of 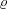 in terms of the distance to the pattern centre PC′, and also determines the size adaptation. In practice it represents the calibration quality of an EBSD system and is therefore a basic prerequisite (Randle, 1992 ▸; Krieger Lassen & Bilde-Sørensen, 1993 ▸; Krieger Lassen, 1994 ▸).

Commonly, PC is roughly known, and despite its imperfections its quality is sufficient for the manual analysis of BKD patterns presented here. However, it may happen that PC is unknown, cannot be extracted unambiguously, or may be incorrect and needs to be confirmed again. For such cases it is desirable to be able to derive a PC position afterwards, *i.e.* preferably without any additional manufacturer-specific information or sophisticated software.

In the past, work has been published (Krieger Lassen, 1999 ▸) which already discussed all available techniques for a derivation of PC. There are procedures which take advantage of the fundamental properties of the gnomonic projection, like shadow techniques (Venables & Bin-Jaya, 1977 ▸; Harland *et al.*, 1981 ▸; Day, 1993 ▸; Mingard *et al.*, 2011 ▸) or the moving-screen technique (Hjelen *et al.*, 1993 ▸), which need access to the instrument used.

The alternative is the utilization of extremely constrained crystal properties in combination with the laws of projective geometry. Unfortunately, seemingly simple analytical approaches in projective geometry are actually very complex and have therefore only been solved numerically (Krieger Lassen & Bilde-Sørensen, 1993 ▸; Basinger *et al.*, 2011 ▸; Wright *et al.*, 2012 ▸; Nolze *et al.*, 2013 ▸, 2018 ▸), *i.e.* due to minimizing the deviation between an experimental and a simulated signal.

Thus, practically all current procedures are unsuitable for deriving the PC *ad hoc*, so an alternative approach is required. Since we only have the gnomonically projected signal, a straightforward solution should be based on projective geometry.

### 3.1. Determination of |**R**|   

For the determination of the position of the PC, the best prerequisite is a BKD pattern of a cubic phase showing easily identifiable poles [*uvw*], *e.g.* 〈001〉, 〈011〉 and 〈111〉, acquired under comparable conditions to the unknown phase. For a cubic phase the symmetry density is quite high, the symmetry directions are easily detectable and lattice parameters are irrelevant. As a matter of fact, any clearly identifiable [*uvw*]_*i*_ of a known phase can be used, *e.g.* the four directions shown in Fig. 4 ▸(*a*).

Moreover, it should be taken into account that the accuracy of the position of a manually derived PC increases when the distance between pole projections is larger and the zone-axis positions are more accurately defined. To reduce positioning errors as much as possible, for the definition of the zone-axis positions the previously briefly explained four-line approach is again recommended [Fig. 4 ▸(*b*)].

The following derivation of PC refers to an analytical solution initially derived for a similar problem in photogrammetry (Killian, 1955*a* ▸,*b* ▸). It uses the intersection of four known zone axes [*uvw*]_*i*_ of a cubic phase with a plane described by the detector screen. In the four-line approach, the considered [*uvw*]_*i*_ do not even have to be visible on the BKD pattern (point *D* in Fig. 1 ▸). The approach can be adapted to non-cubic phases using the general equations for α_*i*_ and β_*i*_ in equation (16).

**Figure 5:**
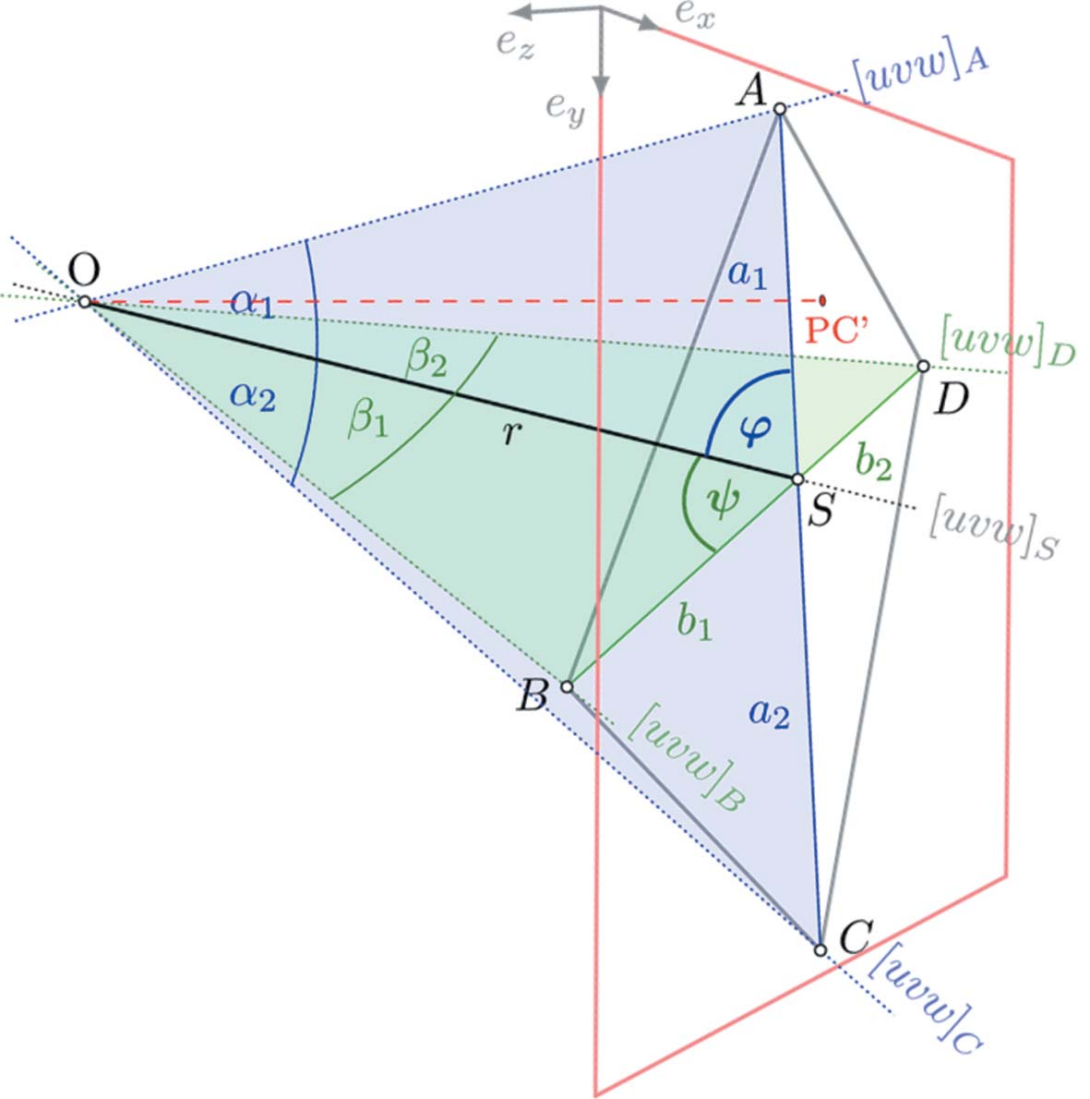
Determination of the projection centre PC 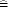 *O* using the intersection of four known zone axes (Killian, 1955*a* ▸,*b* ▸). The unknown angles φ and ψ and the length *r* = |**R**| can be derived from *a*_1_, *a*_2_, *b*_1_, *b*_2_, *b*_2_, α_1_, α_2_, β_1_ and β_2_.

The four [*uvw*]_*A*–*D*_ define two lattice planes (*hkl*), whose intersection point provides a fifth lattice direction [*uvw*]_*S*_

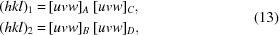


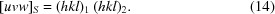
The distances *a*_1_, *a*_2_, *b*_1_ and *b*_2_ between the intersections in the projection plane in Fig. 5 ▸ depend on the projection centre position PC 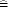 O and the angles between the identified directions [*uvw*]_*i*_: 
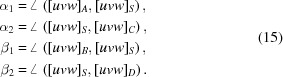
All angles can be easily computed, *e.g.* for a cubic phase by
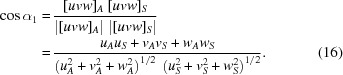
Since 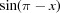 = sin*x*, the sine theorem for plane triangles enables the relationships for *OAS*, *OSC*, *OBS* and *OSD* to be established:
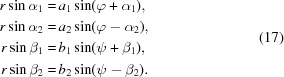
Using the addition theorem 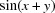 = 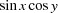 + 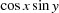, all equations in (17) can be converted to
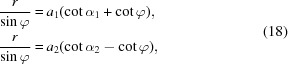


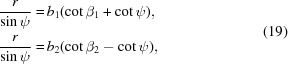
which gives four equations with three unknown variables, *r*, φ and ψ. Equalization of the equations in (18) and (19) enables us to compute φ and ψ from measured distances and calculated angles:
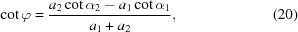


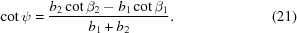
Inserting these into (18) and (19), the required distance *r* = |**R**| can be calculated by
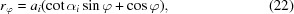


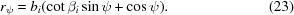
and should actually give the same length. However, in contrast with the expectation of Killian (1955*a* ▸,*b* ▸), the two *r* values are only approximately equal, *i.e.* in general only the following applies:
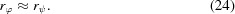
The reason for this is that we consider an exactly defined lattice but measure distances under conditions which are correlated by the four-line approach but not sufficiently constrained that they exactly match the considered crystal metric. However, an adjustment of *a*_*i*_ and *b*_*i*_ by a slight change in the four band positions allows us finally to fulfil the exact condition 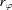 = 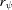.

### 3.2. Determination of PC   

For the final calculation of PC we only need the sub­tetrahedron *O*–*ASB* in Fig. 5 ▸. We can describe the sides of the tetrahedron by the following vectors:
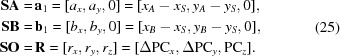
In order to determine the position of *O* = PC, the scalar products of **R**, **a**_1_ and **b**_1_ are helpful and can either be calculated by
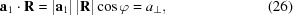


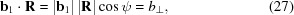
or according to (25) by
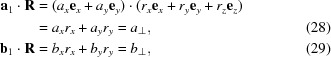
so that for the pattern centre description *r*_*x*_ and *r*_*y*_ result in
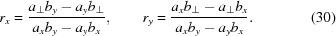
Note that, according to (25), *r*_*x*_ and *r*_*y*_ indicate the deviation from *S* to PC′ in the dimensions used for *a*_*i*_ and *b*_*i*_. For the relative description as used in EBSD systems, these values need to be recalculated and they will depend on the definition of the coordinate system.

The distance between the pattern centre and the projection centre is given by
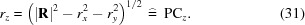
For the BKD patterns of magnetite and schreibersite discussed above, the PC positions have been determined by pattern matching (PM) and the projective-geometry (PG) approach presented here. For the latter, the trace positions were derived using an automated four-line approach, *i.e.* all lines are really derived from the previously defined inter­section points, and the distances and PC were calculated automatically in a computer program. Additionally, for magnetite the manual drawing used already was taken to measure the distances between the five points using a ruler in order to compute PC. Table 3 ▸ contains all the results.

It is encouraging that the two automated techniques deliver nearly identical values. The purely manual solution is still very satisfactory for general discussions of symmetries and angle measurements since the deviations in PC do not affect these results considerably. Our conclusion is that the applied approach is trustworthy for BKD patterns where the PC is not available but a BKD pattern of a known phase collected under comparable geometric conditions exists.

## 4. Summary and conclusions   

Although EBSD has existed as a fully automated technique for decades, the analysis of single patterns is still difficult for unknown phases which have no match in the supplied databases. In such cases, a manual analysis of BKD patterns offers the opportunity to carry out systematic investigations like the correlation of angles between lattice planes and directions or band widths in order to discover relevant indications for a final phase identification. This has been already demonstrated by Martin *et al.* (2017 ▸) for a non-identifiable phase in X-ray diffractograms collected at a synchrotron facility.

Future possible applications include the determination of lattice-parameter ratios, or the identification of the Bravais lattice inclusive lattice parameters, as has been demonstrated already by Li & Han (2015 ▸).

A precondition of a reliable measurement of angles between lattice planes and directions is a correct definition of the lattice-plane traces in a BKD pattern. We have discussed a four-line approach, which consistently fixes the position of all further bands by crystallographic laws.

For a quantitative analysis of the lattice-plane trace positions, the projection-centre position can be derived on the basis of projective geometry. A BKD pattern of a preferably cubic phase, acquired under comparable geometric conditions, delivers the position of the PC via the identification of four zone axes [*uvw*]_*i*_ with known indices.

Finally, we have discussed the use of a classical crystallometric tool, namely the equatorial gnomonic Hilton net, for manual angle measurements in EBSD Kikuchi patterns. The net enables the computation of interplanar angles and the direct measurement of angles between any directions, *e.g.* zone axes, band widths and HOLZ rings. By discussing the underlying crystallographic and geometric conditions, we have provided the theoretical background for improved software-based solutions for the individual analysis of Kikuchi patterns of unknown phases.

## Figures and Tables

**Table 1:** Computation of interplanar angles between cubic lattice planes using their traces manually characterized by slope θ and distance 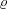 to the pattern centre (in degrees) The indexing of (*hkl*) is only given for comprehensibility, *i.e.* for comparison with ideal angles.

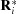		φ_*i*, *j*_ = 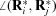 in °, 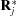					
(*hkl*)	θ	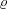	(101)	(011)	( 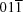 )	( 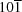 )	( 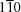 )
(101)	150.1	34.1					
(011)	71.4	43.7	59.7				
( 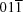 )	315.9	24.3	120.1	90.0			
( 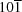 )	251.7	16.6	90.0	119.7	60.2		
( 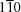 )	16.1	7.2	120.0	60.3	59.9	120.1	
(001)	107.7	11.2	44.9	45.1	135.1	134.8	90.2

**Table 2:** Comparison of measured angles χ using the Hilton net and calculated angles (*a*_0_ = 9.04 Å, *c*_0_ = 4.462 Å) between [*uvw*]_*i*_ defining the cone axes of HOLZ rings in Fig. 4 ▸ The precision of a manual measurement depends on 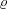 but is commonly not better than 0.5°.

	χ_*i*, *j*_ = 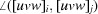					
[*uvw*]	[100]		[101]		[111]	
[101]	26.5°	(26.27°)				
[111]	48.0°	(48.12°)	41.5°	(41.88°)		
[ 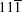 ]	48.0°	(48.12°)	63.0°	(63.07°)	38.5°	(38.48°)

**Table 3:** Determination of the projection-centre position applying pattern matching (PM) (Winkelmann *et al.*, 2018 ▸) and projective geometry (PG) for the BKD patterns displayed in Fig. 3 ▸ (magnetite, cubic) and Fig. 4 ▸ (schreibersite, tetragonal) For magnetite, PC has been also determined completely manually, denoted PG_m_.

Phase	Method	PC_*x*_	PC_*y*_	PC_*z*_
Magnetite	PM	0.493	0.279	0.786
	PG	0.491	0.281	0.785
	PG_m_	0.48	0.27	0.78
Schreibersite	PM	0.474	0.116	0.704
	PG	0.474	0.118	0.705

## Footnotes

1 Actually an infinite number of (*hkl*)_*i*_.

2 The length of vector **x** in Fig. 1 ▸ is unconventionally multiplied by the aspect ratio *A* of the image, *i.e.* equal pixel shifts deliver different ΔPC_*x*_ = ΔPC_*y*_/*A*.

3 For different manufacturers, top and bottom are exchanged by a rotation around **x** at 0.5**y** so that (PC_*x*_, PC_*y*_, PC_*z*_) → (PC_*x*_, 1 − PC_*y*_, −PC_*z*_).

4 For the precision of 0.1° the spin animation in *PowerPoint* was used.
